# On Leader-Following Consensus in Multi-Agent Systems with Discrete Updates at Random Times

**DOI:** 10.3390/e22060650

**Published:** 2020-06-12

**Authors:** Ricardo Almeida, Ewa Girejko, Snezhana Hristova, Agnieszka Malinowska

**Affiliations:** 1 Center for Research and Development in Mathematics and Applications, Department of Mathematics, University of Aveiro, 3810-193 Aveiro, Portugal; ricardo.almeida@ua.pt; 2 Faculty of Computer Science, Bialystok University of Technology, 15-351 Białystok, Poland; a.malinowska@pb.edu.pl; 3 Faculty of Mathematics and Computer Science, University of Plovdiv Paisii Hilendarski, 4027 Plovdiv, Bulgaria; snehri@gmail.com

**Keywords:** multi-agent system, communications at discrete random times, leader-following consensus, exponential distribution, differential equations with impulses

## Abstract

This paper studies the leader-following consensus problem in continuous-time multi-agent networks with communications/updates occurring only at random times. The time between two consecutive controller updates is exponentially distributed. Some sufficient conditions are derived to design the control law that ensures the leader-following consensus is asymptotically reached (in the sense of the expected value of a stochastic process). The numerical examples are worked out to demonstrate the effectiveness of our theoretical results.

## 1. Introduction

In recent years, we witnessed increasing attention on the distributed cooperative control of dynamic multi-agent systems due to their vast applications in various fields. In many situations, groups of dynamic agents need to interact with each other and their goal is to reach an agreement (consensus) on a certain task. For example, it can be flocking of birds during migration [1,2] to eventually reach their destinations; or robot teams synchronized in order to accomplish their collective tasks [3,4]. The main challenge for distributed cooperative control of multi-agent systems is that interaction between agents is only based on local information. There already exists a vast literature concerning first-order [3,5], second-order [6,7], and fractional-order [8,9,10] networks. For a survey of the recent results, we refer the reader to [11]. Within different approaches to the consensus problem in multi-agent networks, one can find continuous-time agents’ state evolving (the state trajectory is a continuous curve) [3,5,12,13], discrete-time agents’ state evolving (the state trajectory is a sequence of values) [14,15,16,17,18,19], and both continuous and discrete-time agents’ state evolving (the domain of the state trajectory is any time scale) [20,21,22,23]. An important question connected with the consensus problem is whether the communication topology is fixed over time or is time-varying; that is, communications channels are allowed to change over time [24]. The latter case seems to be more realistic; therefore, scientists mostly focus on it. Going farther, in real-word situations, it may happen that agents’ states are continuous but an exchange of information between agents occurs only at discrete time instants (update times). This issue was already addressed in the literature [25]. In this paper we also investigate such a situation. However, our approach is new and more challenging: we consider the case when agents exchange information between each other at random instants of times. Another question to be answered is whether the consensus problem is considered with or without the leader. Based on the existence of a leader, there are two kinds of consensus problems: leaderless consensus and leader-following consensus. This last-mentioned problem relies on establishing conditions under which, through local interactions, all the agents reach an agreement upon a common state (consensus), which is defined by the dynamic leader. A great number of works have been already devoted to the consensus problem with the leader (see, e.g., [24,26,27,28] and the references given there).

In the present paper, we investigate a leader-following consensus for multi-agent systems. It is assumed that the agents’ state variables are continuous but exchange information between them occurs only at discrete time instants (update times) appearing randomly. In other words, the consensus control law is applied at those update times. We analyze the case when a sequence of update times is a sequence of random variables and the waiting time between two consecutive updates is exponentially distributed. To avoid unnecessary complexity, we assume that the update times are the same for all agents. Combining of continuity of state variables with discrete random times of communications requires the introduction of an artificial state variable for each agent that evolves in continuous-time and is allowed to have discontinuities; the primary state variable is continuous for all time. Between update times, both the original state and the artificial variable evolve continuously according to some specified dynamics. At randomly occurring update times, the state variable keeps its current value, while the artificial variable is updated by the state values received from other agents, including the leader. It is worth noting that, in the case of deterministic update times, known initially, the idea of artificial state variables is applied in [15,16,25]. The presence of randomly occurring update times in the model leads to a total change of the behavior of the solutions. They are changed from deterministic real valued functions to stochastic processes. This requires combining of results from the probability theory with the ones of the theory of ordinary differential equations with impulses. In order to analyze the leader-following consensus problem, we define the state error system and a sample path solution of this system. Since solutions to the studied model of a multi-agent system with discrete-time updates at random times are stochastic processes, asymptotically reaching leader-following consensus is understood in the sense of the expected value.

The paper is organized in the following manner. In Section 2, we describe the multi-agent system of our interest in detail. Some necessary definitions, lemmas, and propositions from probability theory are given in Section 3. Section 4 contains our main results. First, we describe a stochastic process that is a solution to the continuous-time system with communications at random times. Next, sufficient conditions for the global asymptotic leader-following consensus in a continuous-time multi-agent system with discrete-time updates occurring at random times are proven. In Section 5, an illustrative example with numerical simulations is presented to verify theoretical discussion. Some concluding remarks are drawn in Section 6.

Notation: For a given vector $x\in R^{n}$, ∥x∥ stands for its Euclidean norm $∥x∥=\sqrt{x^{T}x}$. For a given square n×n matrix, $A=[a_{ij}]$, ∥A∥ stands for its spectral norm $∥A∥=\sqrt{\max_{i}\left\{\lambda_{i}\right\}}$, where $\lambda_{i}$ are the eigenvalues of $A.A^{T}$. We have $∥A∥\leq n\max_{i,j}\left|a_{ij}\right|$ and $∥e^{A}∥\leq e^{∥A∥}$.

## 2. Statement of the Model

We consider a multi-agent system consisting of *N* agents and one leader. The state of agent *i* is denoted by $y_{i}:\left[t_{0},\infty \right)\to R$, i=1,…,N, and the state of the leader by $y_{r}:\left[t_{0},\infty \right)\to R$, where $t_{0}\geq 0$ is a given initial time. Without information exchange between agents, the leader has no influence on the other agents (see Example 1, Case 1.1, and Example 2, Case 2.1). In order to analyze the influence of the leader on the behavior of the other agents, we assume that there is information exchange between agents but it occurs only at random update times. In other words, the model is set up as the continuous-time multi-agent system with discrete-time communications/updates occurring only at random times.

Let us denote by (Ω,F,P) a probability space, where Ω is the sample space, F is a σ-algebra on Ω, and *P* is the probability on F. Consider a sequence of independent, exponentially-distributed random variables ${\left\{\tau_{k}\right\}}_{k=1}^{\infty }$ with parameter λ>0 and such that $\sum_{k=1}^{\infty }\tau_{k}=\infty$ with a probability 1. Define the sequence of random variables ${\left\{\xi_{k}\right\}}_{k=0}^{\infty }$ by

(1) $$\xi_{0}=t_{0},\;\xi_{k}=t_{0}+\sum_{i=1}^{k}\tau_{i},k=1,2,\ldots ,$$

where $t_{0}$ is a given initial time. The random variable $\tau_{k}$ measures the waiting time of the *k*-th event time after the (k−1)-st controller update occurs and the random variable $\xi_{k}$ is connected with the random event time and it denotes the length of time until *k* controller updates occur for $t\geq t_{0}$. At each time $\xi_{k}$ agent *i* updates its state variable according to the following equation:

$$\Delta y_{i}\left(\xi_{k}\right)=u_{i}\left(\xi_{k}\right)\;,\;\;i=1,\ldots ,N\;,\;\;k=1,2,\ldots ,$$

where $u_{i}:R\to R$ is the control input function for the *i*-th agent. Here, $\Delta y_{i}\left(\xi_{k}\right)$ is the difference between the value of the state variable of the *i*-th agent after the update $y_{i}\left(\xi_{k}+0\right)$ and before it $y_{i}\left(\xi_{k}\right)$; i.e., $\Delta y_{i}\left(\xi_{k}\right)=y_{i}\left(\xi_{k}+0\right)-y_{i}\left(\xi_{k}\right)$. The state of the leader remains unchanged; that is,

$$\Delta y_{r}\left(\xi_{k}\right)=0.$$

For each agent *i* we consider the control law, at the random times $\xi_{k},\;k=1,2,\ldots$, based on the information it receives from its neighboring agents and the leader:

$$u_{i}\left(\xi_{k}\right)=-\left[\sum_{j=1}^{N}a_{ij}\left(\tau_{k}\right)\left(y_{i}\left(\xi_{k}\right)-y_{j}\left(\xi_{k}\right)\right)+\omega_{i}\left(\tau_{k}\right)\left(y_{i}\left(\xi_{k}\right)-y_{r}\left(\xi_{k}\right)\right)\right],\;\;k=1,2,\ldots \;,$$

where weights $a_{ii}\left(t\right)\equiv 0,i=1,2\ldots ,N,$ and $a_{ij}\left(t\right)\geq 0,\;t\geq t_{0},\;i,j=1,2,\ldots ,N,$ are entries of the weighted connectivity matrix A(t) at time *t*:

$$A\left(t\right)=\left[\begin{array}{ccccc} 0 & a_{12}\left(t\right) & a_{13}\left(t\right) & \ldots & a_{1N}\left(t\right)\\ a_{21}\left(t\right) & 0 & a_{23}\left(t\right) & \ldots & a_{2N}\left(t\right)\\ \ldots & \ldots & \ldots & \ldots & \ldots \\ a_{N1}\left(t\right) & a_{N2}\left(t\right) & a_{N3}\left(t\right) & \ldots & 0 \end{array}\right],$$

and $\omega_{i}\left(t\right)>0$ if the virtual leader is available to agent *i* at time *t*, while $\omega_{i}\left(t\right)=0$ otherwise. Between two update times $\xi_{k-1}$ and $\xi_{k}$, any agent *i* has information only about his own state. More precisely, the dynamics of agent *i* are described by

$$\begin{array}{c} y_{i}^{\prime }\left(t\right)=-\left(b_{i}\left(\tau_{k}\right)-c_{i}\left(\tau_{k}\right)\right)y_{i}\left(t\right),\;\;\mathrm{for}\;t\in \left(\xi_{k-1},\xi_{k}\right],\;\;\;i=1,2,\ldots ,N,\;\;k=1,2,\ldots , \end{array}$$

where $b_{i}\in C\left(\left[t_{0},\infty \right),\left(0,\infty \right)\right),\;c_{i}\in C\left(\left[t_{0},\infty \right),\left[0,\infty \right)\right)$, i=1,…,N.

The leader for the multi-agent system is an isolated agent with constant reference state

$$\begin{array}{c} y_{r}^{\prime }\left(t\right)=0. \end{array}$$

Observe that the model described above can be written as a system of differential equations with impulses at random times $\xi_{k},\;k=1,2,\ldots$, and waiting time between two consecutive updates $\tau_{k}$ as follows:

(2) $$\begin{array}{cc} \; & y_{r}^{\prime }\left(t\right)=0\;\;\;\mathrm{for}\;t\in \left(\xi_{k-1},\xi_{k}\right],\\ \; & y_{i}^{\prime }\left(t\right)=-\left(b_{i}\left(\tau_{k}\right)-c_{i}\left(\tau_{k}\right)\right)y_{i}\left(t\right)\;\;\;\mathrm{for}\;t\in \left(\xi_{k-1},\xi_{k}\right],\\ \; & \Delta y_{r}\left(\xi_{k}\right)=0,\\ \; & \Delta y_{i}\left(\xi_{k}\right)=-\left[\sum_{j=1}^{N}a_{ij}\left(\tau_{k}\right)\left(y_{i}\left(\xi_{k}\right)-y_{j}\left(\xi_{k}\right)\right)+\omega_{i}\left(\tau_{k}\right)\left(y_{i}\left(\xi_{k}\right)-y_{r}\left(\xi_{k}\right)\right)\right],\\ \; & \;\;\;\;\;\;\;k=1,2,\ldots \;,\;i=1,\ldots ,N, \end{array}$$

with initial conditions

(3) $$y_{r}\left(t_{0}\right)=y_{r}^{0},\;\;\;y_{i}\left(t_{0}\right)=y_{i}^{0},\;\;i=1,2,\ldots ,N.$$

We introduce an additional (artificial) variable $Y_{i}$ for each state $y_{i},\;i=1,2,\ldots ,N$, such that it has discontinuities at random times $\xi_{k}$ and $Y_{r}=y_{r}$. These variables allow us to keep the state of each agent $y_{i},\;i=1,2,\ldots ,N,$ as a continuous function of time. Between two update times $\xi_{k-1}$ and $\xi_{k}$ the evolution of $Y_{i}$ and $Y_{r}$ are given by

(4) $$Y_{i}^{\prime }\left(t\right)=\left(b_{i}\left(\tau_{k}\right)-c_{i}\left(\tau_{k}\right)\right)Y_{i}\left(t\right),\;i=1,2,\ldots ,N,\;\;\;\;\;Y_{r}^{\prime }\left(t\right)=0.$$

Then, by Equations (2) and (4), we obtain

$$\begin{array}{cc} y_{i}^{\prime }\left(t\right)+Y_{i}^{\prime }\left(t\right) & =-\left(b_{i}\left(\tau_{k}\right)-c_{i}\left(\tau_{k}\right)\right)y_{i}\left(t\right)+\left(b_{i}\left(\tau_{k}\right)-c_{i}\left(\tau_{k}\right)\right)Y_{i}\left(t\right)\\ \; & =-b_{i}\left(\tau_{k}\right)\left(y_{i}\left(t\right)-Y_{i}\left(t\right)\right)+c_{i}\left(\tau_{k}\right)\left(y_{i}\left(t\right)-Y_{i}\left(t\right)\right),\;i=1,\ldots ,N. \end{array}$$

Consequently, we get the following system:

(5) $$\begin{array}{cc} \; & y_{i}^{\prime }\left(t\right)=-b_{i}\left(\tau_{k}\right)\left(y_{i}\left(t\right)-Y_{i}\left(t\right)\right),\;\;i=1,\ldots ,N,\\ \; & Y_{i}^{\prime }\left(t\right)=c_{i}\left(\tau_{k}\right)\left(y_{i}\left(t\right)-Y_{i}\left(t\right)\right),\;\;i=1,\ldots ,N,\\ \; & y_{r}^{\prime }\left(t\right)=0,\;\;\;\;\;\;Y_{r}^{\prime }\left(t\right)=0\;\;\;\;\;\;\;\;\;\;\;\;\;\;\;\;\;\;\;\;\;\;\;\;\;\;\;\;\;\;\mathrm{for}\;\;t\in \left(\xi_{k-1},\xi_{k}\right],\;\;k=1,2\ldots . \end{array}$$

At each update time we set:

(6) $$\begin{array}{cc} \; & y_{i}\left(\xi_{k}+0\right)=y_{i}\left(\xi_{k}\right),\;i=1,\ldots ,N,\;\;\;k=1,2,\ldots ,\\ \; & Y_{i}\left(\xi_{k}+0\right)=-\left[\sum_{j=1}^{N}a_{ij}\left(\tau_{k}\right)\left(Y_{i}\left(\xi_{k}\right)-y_{j}\left(\xi_{k}\right)\right)+\omega_{i}\left(\tau_{k}\right)\left(Y_{i}\left(\xi_{k}\right)-y_{r}\left(\xi_{k}\right)\right)\right]+Y_{i}\left(\xi_{k}\right),\\ \; & \;\;\;\;\;\;\;\;\;\;\;\;\;\;\;\;\;\;\;\;\;\;\;\;\;\;\;\;\;\;\;\;\;\;\;\;\;\;\;\;\;\;\;\;\;\;\;\;\;\;\;\;\;\;\;\;\;i=1,\ldots ,N,\;\;k=1,2,\cdots ,\\ \; & y_{r}\left(\xi_{k}+0\right)=y_{r}\left(\xi_{k}\right),\;\;Y_{r}\left(\xi_{k}+0\right)=Y_{r}\left(\xi_{k}\right),\;\;\;\;\;k=1,2,\ldots . \end{array}$$

The initial conditions for (5) and (6) are:

(7) $$y_{r}\left(t_{0}\right)=y_{r}^{0},\;\;\;y_{i}\left(t_{0}\right)=y_{i}^{0},\;\;i=1,2,\ldots ,N,\;\;\;Y_{i}\left(t_{0}\right)=y_{r}^{0},\;\;i=1,2,\ldots ,N,\;\;Y_{r}\left(t_{0}\right)=y_{r}^{0}.\;$$

Observe that dynamics described by (5) lead to a decrease of the absolute difference between a state variable $y_{i}$ and an artificial variable $Y_{i}$, i=1,2,…,N. Whereas by (6), the value of $Y_{i}$ is updated using the information received, while $y_{i}$ remains unchanged. Therefore, Equations (5) and (6) provide a formal description of the multi-agent system with continuous-time states of agents and information exchange between agents occurring at discrete-time instants.

Let $x_{i}\left(t\right):=y_{i}\left(t\right)-y_{r}\left(t\right),\;\;X_{i}\left(t\right):=Y_{i}\left(t\right)-y_{r}\left(t\right),\;i=1,2,\ldots ,N,$ be errors between any state $y_{i}$ or $Y_{i}$, and the leader state $y_{r}$, at time *t*. Then, by (5)–(7), one gets the following error system:

(8) $$\begin{array}{cc} \; & x_{i}^{\prime }\left(t\right)=-b_{i}\left(\tau_{k}\right)\left(x_{i}\left(t\right)-X_{i}\left(t\right)\right),\;i=1,\ldots ,N,\\ \; & X_{i}^{\prime }\left(t\right)=c_{i}\left(\tau_{k}\right)\left(x_{i}\left(t\right)-X_{i}\left(t\right)\right),\;i=1,\ldots ,N,\;\;\;\;\;\;\;\mathrm{for}\;\;t\in \left(\xi_{k-1},\xi_{k}\right],\\ \; & x_{i}\left(\xi_{k}+0\right)=x_{i}\left(\xi_{k}\right),\;\;i=1,2,\ldots ,N,\\ \; & X_{i}\left(\xi_{k}+0\right)=d_{ii}\left(\tau_{k}\right)X_{i}\left(\xi_{k}\right)+\sum_{j=1}^{N}d_{ij}\left(\tau_{k}\right)x_{j}\left(\xi_{k}\right)+X_{i}\left(\xi_{k}\right),\\ \; & \;\;\;\;\;\;\;\;\;\;\;\;\;\;\;\;\;\;\;\;\;\;\;\;\;i=1,2,\ldots ,N,\;\;\;k=1,2,\ldots ,\\ \; & x_{i}\left(t_{0}\right)=y_{i}^{0}-y_{r}^{0},\;\;i=1,2,\ldots ,N,\;\;\;X_{i}\left(t_{0}\right)=0,\;\;i=1,2,\ldots ,N, \end{array}$$

where coefficients $d_{ij}$ are the entries of the matrix

$$\tilde{D}\left(t\right)=\left[\begin{array}{cccc} -\sum_{j=1}^{N}a_{1j}\left(t\right)-\omega_{1}\left(t\right) & a_{12}\left(t\right) & \ldots & a_{1N}\left(t\right)\\ a_{21}\left(t\right) & -\sum_{j=1}^{N}a_{2j}\left(t\right)-\omega_{2}\left(t\right) & \ldots & a_{2N}\left(t\right)\\ \ldots & \ldots & \ldots & \ldots \\ a_{N1}\left(t\right) & a_{N2}\left(t\right) & \ldots & -\sum_{j=1}^{N}a_{Nj}\left(t\right)-\omega_{N}\left(t\right) \end{array}\right],$$

i.e.,

$$\begin{array}{cc} d_{ij}\left(t\right) & =a_{ij}\left(t\right),\;i\neq j,i,j=1,2,\ldots ,N,\\ d_{ii}\left(t\right) & =-\sum_{j=1}^{N}a_{ij}\left(t\right)-\omega_{i}\left(t\right),\;i=1,2,\ldots ,N. \end{array}$$

Now let us introduce the 2N×2N-dimensional matrices

$$C\left(t\right)=\left[\begin{array}{ccccccc} -b_{1}\left(t\right) & b_{1}\left(t\right) & 0 & 0 & \ldots & 0 & 0\\ c_{1}\left(t\right) & -c_{1}\left(t\right) & 0 & 0 & \ldots & 0 & 0\\ 0 & 0 & -b_{2}\left(t\right) & b_{2}\left(t\right) & \ldots & 0 & 0\\ 0 & 0 & c_{2}\left(t\right) & -c_{2}\left(t\right) & \ldots & 0 & 0\\ \ldots & \ldots & \ldots & \ldots & \ldots & \ldots & \ldots \\ 0 & 0 & \ldots & 0 & 0 & -b_{N}\left(t\right) & b_{N}\left(t\right)\\ 0 & 0 & \ldots & 0 & 0 & c_{N}\left(t\right) & -c_{N}\left(t\right) \end{array}\right],$$

and

$$D\left(t\right)=\left[\begin{array}{ccccccccc} 0 & 0 & 0 & 0 & 0 & 0 & \ldots & 0 & 0\\ 0 & d_{1,1}\left(t\right)+1 & d_{1,2}\left(t\right) & 0 & d_{1,3}\left(t\right) & 0 & \ldots & d_{1,N}\left(t\right) & 0\\ 0 & 0 & 0 & 0 & 0 & 0 & \ldots & 0 & 0\\ d_{2,1}\left(t\right) & 0 & 0 & d_{2,2}\left(t\right)+1 & d_{2,3}\left(t\right) & 0 & \ldots & d_{2,N}\left(t\right) & 0\\ \ldots & \ldots & \ldots & \ldots & \ldots & \ldots & \ldots \\ 0 & 0 & 0 & 0 & 0 & 0 & \ldots & 0 & 0\\ d_{N,1}\left(t\right) & 0 & d_{N,2}\left(t\right) & 0 & d_{N,2}\left(t\right) & 0 & \ldots & 0 & d_{N,N}\left(t\right)+1 \end{array}\right].$$

Then, denoting $Z={\left(x_{1},X_{1},x_{2},X_{2}\ldots ,x_{N},X_{N}\right)}^{T}$, we can write error system (8) in the following matrix form:

(9) $$\begin{array}{cc} \; & Z^{\prime }\left(t\right)=C\left(\tau_{k}\right)Z\left(t\right),\;\;\;\;\mathrm{for}\;\;t\in \left(\xi_{k-1},\xi_{k}\right],\;k=1,2,\ldots ,\\ \; & Z\left(\xi_{k}+0\right)=D\left(\tau_{k}\right)Z\left(\xi_{k}\right),\;k=1,2,\ldots ,\\ \; & Z\left(t_{0}\right)=Z_{0}, \end{array}$$

where $Z_{0}={\left(x_{1}^{0},0,x_{2}^{0},0,\ldots ,x_{N}^{0},0\right)}^{T}\in R^{2N},\;\;\;x_{i}^{0}=y_{i}^{0}-y_{r}^{0},\;i=1,2,\ldots ,N.$

## 3. Some Preliminary Results from Probability Theory

In this section, having in mind the definitions of random variables ${\left\{\tau_{k}\right\}}_{k=1}^{\infty }$ and ${\left\{\xi_{k}\right\}}_{k=1}^{\infty }$, given in Section 2, we list some facts from probability theory that will be used in the proofs of our main results.

**Proposition** **1** ([29]). *The random variable $\Xi =\sum_{i=1}^{k}\tau_{i}$ is Erlang distributed with a probability density function $f_{\Xi }\left(t\right)=\lambda e^{-\lambda t}\frac{{\left(\lambda t\right)}^{k-1}}{(k-1)!}$ and cumulative distribution function $F\left(t\right)=P\left(\Xi <t\right)=1-e^{-\lambda t}\sum_{j=0}^{k-1}\frac{{\left(\lambda t\right)}^{j}}{j!}$.*

Let $t\geq t_{0}$ be a fixed point. Consider the events

$$S_{k}\left(t\right)=\left\{\omega \in \Omega :\;\xi_{k-1}\left(\omega \right)<t<\xi_{k}\left(\omega \right)\right\},\;\;\;k=1,2,\ldots$$

and define the stochastic processes $\Delta_{k}\left(t\right)$, k=1,2,…, by

$$\begin{array}{c} \Delta_{k}\left(t\right)=\left\{\begin{array}{c} 1\;\;\;\;\mathrm{for}\;\;\;\;\omega \in S_{k}\left(t\right)\\ 0\;\;\;\;\mathrm{for}\;\;\;\;\omega \notin S_{k}\left(t\right) \end{array}\right.. \end{array}$$

Note that, for any fixed point *t* and any element ω∈Ω, there exists a natural number *k* such that $\omega \in S_{k}\left(t\right)$ and $\omega \notin S_{j}\left(t\right)$ for j≠k, or for any fixed point *t* there exists a natural number *k* such that $\Delta_{k}\left(t\right)=1$ and $\Delta_{j}\left(t\right)=0$ for j≠k.

**Lemma** **1** ([30] Lemma 2.1). *Let ${\left\{\tau_{k}\right\}}_{k=1}^{\infty }$ be independent, exponentially-distributed random variables with a parameter λ and $\xi_{k}=t_{0}+\sum_{i=1}^{k}\tau_{i}$. Then,*

$$E\left(\Delta_{k}\left(t\right)\right)=\frac{\lambda^{k}{\left(t-t_{0}\right)}^{k}}{k!}e^{-\lambda (t-t_{0})},\;\;for\;\;\;t\geq t_{0}\;\;and\;\;k=1,2,\ldots ,$$

*where E{.} denotes the mathematical expectation.*

**Corollary** **1** ([30]). *The probability that there will occur exactly k controller updates of each agent until the time t, $t\geq t_{0}$, is given by the equality*

$$P\left(S_{k}\left(t\right)\right)=\frac{\lambda^{k}{\left(t-t_{0}\right)}^{k}}{k!}e^{-\lambda (t-t_{0})}.$$

**Definition** **1** ([29]). *We say that the stochastic processes m and n satisfy the inequality m(t)≤n(t), for t∈J⊂R, if the state space of the stochastic processes v(t)=m(t)−n(t) is (−∞,0].*

**Proposition** **2** ([29]). *If the stochastic processes y and u satisfy the inequality m(t)≤n(t) for t∈J⊂R, then E(m(t))≤E(n(t)) for t∈J*.

**Proposition** **3** ([29]). *Let a>0 be a real constant and τ be an exponentially-distributed random variable with parameter λ>a. Then, $E\left(e^{a\tau }\right)=\frac{\lambda }{\lambda -a}$*.

## 4. Leader-Following Consensus

Consider the sequence of points ${\left\{t_{k}\right\}}_{k=1}^{\infty }$, where the point $t_{k}$ is an arbitrary value of the corresponding random variable $\tau_{k},\;k=1,2,\ldots$. Define the increasing sequence of points ${\left\{T_{k}\right\}}_{k=0}^{\infty }$ by $T_{0}=t_{0}$ and $T_{k}=T_{0}+\sum_{j=1}^{k}t_{j}$ for k=1,2,….

**Remark** **1.** *Note that if $t_{k}$ is a value of the random variable $\tau_{k}$, k=1,2,…, then $T_{k}$ is a value of the random variable $\xi_{k}$, k=1,2,…, correspondingly.*

Since the multi-agent system with the leader described by system (2)–(3) is equivalent to system (9), we focus on initial value problem (9).

Let us consider the following system of impulsive differential equations with fixed points of impulses and fixed length of action of the impulses:

(10) $$\begin{array}{c} x_{i}^{\prime }\left(t\right)=-b_{i}\left(t_{k}\right)\left(x_{i}\left(t\right)-X_{i}\left(t\right)\right),\;\mathrm{for}\;t\in \left(T_{k-1},T_{k}\right],\\ X_{i}^{\prime }\left(t\right)=c_{i}\left(t_{k}\right)\left(x_{i}\left(t\right)-X_{i}\left(t\right)\right),\;\mathrm{for}\;t\in \left(T_{k-1},T_{k}\right],\\ x_{i}\left(T_{k}+0\right)=x_{i}\left(T_{k}\right),\\ X_{i}\left(T_{k}+0\right)=\left(1+d_{ii}\left(t_{k}\right)\right)X_{i}\left(T_{k}\right)+\sum_{j=1}^{N}d_{ij}\left(t_{k}\right)x_{j}\left(T_{k}\right),\\ x_{i}\left(t_{0}\right)=x_{i}^{0},\;X_{i}\left(t_{0}\right)=0,\;k=1,2,\ldots ,\;i=1,2,\ldots ,N \end{array}$$

or its equivalent matrix form

(11) $$\begin{array}{c} Z^{\prime }\left(t\right)=C\left(t_{k}\right)Z\left(t\right),\;\mathrm{for}\;t\in \left(T_{k-1},T_{k}\right],\\ Z\left(T_{k}+0\right)=D\left(t_{k}\right)Z\left(T_{k}\right),\;k=1,2,\ldots ,\\ Z\left(t_{0}\right)=Z_{0}\;. \end{array}$$

Note that system (11) is a system of impulsive differential equations with impulses at deterministic time moments ${\left\{T_{k}\right\}}_{k=0}^{\infty }$. For a deeper discussion of impulsive differential equations we refer the reader to [31] and the references given there. The solution to (11) depends not only on the initial condition $\left(t_{0},Z_{0}\right)$ but also on the moments of impulses $T_{k},\;k=1,2,\ldots$, i.e., on the arbitrary chosen values $t_{k}$ of the random variables $\tau_{k},\;k=1,2,\ldots$, and is given by

$$Z\left(t;t_{0},Z_{0},\left\{T_{k}\right\}\right)=e^{C\left(t_{k}\right)\left(t-T_{k-1}\right)}Z_{0}\prod_{i=1}^{k-1}\left(D\left(t_{k-i}\right)e^{C\left(t_{k-i}\right)t_{k-i}}\right),\;t\in \left(T_{k-1},T_{k}\right],\;k=1,2,\ldots .$$

The set of all solutions $Z(t;t_{0},Z_{0},\left\{T_{k}\right\})$ of the initial value problems of type (11) for any values $t_{k}$ of the random variables $\tau_{k},\;k=1,2,\ldots$, generates a stochastic process with state space $R^{2N}$. We denote it by $Z(t;t_{0},Z_{0},\left\{\tau_{k}\right\})$ and call it a solution to initial value problem (9). Following the ideas of a sample path of a stochastic process [29,32] we define a sample path solution of studied system (9).

**Definition** **2.** *For any given values $t_{k}$ of the random variables $\tau_{k}$, k=1,2,3,…, respectively, the solution $Z(t;t_{0},Z_{0},\left\{T_{k}\right\})$ of the corresponding initial value problem (10) is called a sample path solution of initial value problem (9).*

**Definition** **3.** *A stochastic process $Z(t;t_{0},Z_{0},\left\{\tau_{k}\right\})$ with an uncountable state space $R^{2N}$ is said to be a solution of initial value problem (9) if, for any values $t_{k}$ of the random variables $\tau_{k},\;k=1,2,\ldots$, the corresponding function $Z(t;t_{0},Z_{0},\left\{T_{k}\right\})$ is a sample path solution of initial value problem (9).*

Let the stochastic process $Z\left(t;t_{0},Z_{0},\left\{\tau_{k}\right\}\right),\;Z={\left(x_{1},X_{1},x_{2},X_{2},\ldots ,x_{N},X_{N}\right)}^{T},$ with an uncountable state space $R^{2N}$ be a solution of initial value problem with random impulses (9).

**Definition** **4.** *We say that the leader-following consensus is reached asymptotically in multi-agent system (2) if, for any $t_{0}\geq 0$ and any $y^{0}\in R^{N}$,*

(12) $$\lim_{t\to \infty }E\left|y_{i}\left(t;t_{0},y^{0},\left\{\tau_{k}\right\}\right)-y_{r}\left(t;t_{0},y^{0},\left\{\tau_{k}\right\}\right)\right|=0,\;\;\;for\;i=1,2,\ldots ,N,$$

*where $y^{0}={\left(y_{1}^{0},y_{2}^{0},\ldots ,y_{N}^{0},y_{r}^{0}\right)}^{T}$.*

**Remark** **2.** *Observe that since $x_{i}\left(t\right)=y_{i}\left(t\right)-y_{r}\left(t\right)$, i=1,…,N, and initial value problem (2)–(3) is equivalent to initial value problem (9), equality (12) means that*

*$\lim_{t\to \infty }E∥x\left(t;t_{0},x^{0},\left\{\tau_{k}\right\}\right)∥=0$, where $x^{0}={\left(x_{1}^{0},...,x_{N}^{0}\right)}^{T}$.*

Now we prove the main results of the paper, which are sufficient conditions for the leader-following consensus in a continuous-time multi-agent system with discrete-time updates occurring at random times.

**Theorem** **1.** *Assume that:*

**(A1)**  *The inequalities*
  (13) $$0<b_{i}\left(t\right)<1,\;0\leq c_{i}\left(t\right)\leq 1,\;\;\;\;\;for\;\;\;\;t\geq t_{0},\;i=1,2\ldots ,N,$$
  *hold, and there exists a real α∈(0,1) such that*
  (14) $$|1-\sum_{j=1}^{N}a_{ij}\left(t\right)-\omega_{i}\left(t\right)|<\frac{\alpha }{2N}\;\;\;for\;\;t\geq t_{0},\;i=1,2\ldots ,N,$$
  *and*
  (15) $$0\leq a_{ij}\left(t\right)<\frac{\alpha }{2N}\;\;for\;\;t\geq t_{0},\;i,j=1,2\ldots ,N,\;i\neq j.$$
**(A2)**  *The random variables $\tau_{k},\;\;k=1,2,\ldots$, are independently exponentially distributed with the parameter λ such that $\lambda >\frac{2N}{1-\alpha }$.*

*Then, for any initial point $t_{0}\geq 0$ the solution $Z(t;t_{0},Z_{0},\left\{\tau_{k}\right\})$ of the initial value problem with random moments of impulses (9) is given by the formula*

(16) $$\begin{array}{cc} Z(t;t_{0},Z_{0},\left\{\tau_{k}\right\}) & =e^{C\left(t_{k}\right)\left(t-\xi_{k}\right)}\left(\prod_{i=1}^{k-1}\left(D\left(\tau_{k-i}\right)e^{C\left(\tau_{k-i}\right)\tau_{k-i}}\right)\right)Z_{0}\\ \; & for\;\;t\in (\xi_{k-1},\xi_{k}],\;k=1,2,\ldots \;, \end{array}$$

*and the expected value of the solution satisfies the inequality*

$$E\left(∥Z\left(t;t_{0},Z_{0},\left\{\tau_{k}\right\}\right)∥\right)\leq ∥Z_{0}∥e^{\left(2N+\alpha \lambda -\lambda \right)\left(t-t_{0}\right)}.$$

**Proof.**  Let $t_{0}\geq 0$ be an arbitrary given initial time. According to (A1), we have

$$∥C\left(t\right)∥\leq 2N\max_{i=1,2,\ldots ,N}\left\{b_{i}\left(t\right),c_{i}\left(t\right)\right\}\leq 2N,$$

$$∥D\left(t\right)∥\leq 2N\max\left\{|1+d_{ii}\left(t\right)|,\max_{i,j=1,2,\ldots ,N,\;i\neq j}\left\{\right|d_{i,j}\left(t\right)\left|\right\}\right\}<\alpha ,$$

and $∥e^{C(t)}∥\leq e^{2N}$ for $t\geq t_{0}$. For any k=1,2,…, we choose an arbitrary value $t_{k}$ of the random variable $\tau_{k}$ and define the increasing sequence of points $T_{0}=t_{0}$, $T_{k}=t_{0}+\sum_{j=1}^{k}t_{j},\;\;k=1,2,3,\ldots$. By Remark 1, for any natural *k*, $T_{k}$ is a value of the random variable $\xi_{k}$. Consider the initial value problem of impulsive differential equations with fixed points of impulses (11). The solution of initial value problem (11) is given by the formula

$$\begin{array}{cc} Z(t;t_{0},Z_{0},\left\{T_{k}\right\})\; & =e^{C\left(t_{k}\right)\left(t-T_{k-1}\right)}\left(\prod_{i=1}^{k-1}\left(D\left(t_{k-i}\right)e^{C\left(t_{k-i}\right)t_{k-i}}\right)\right)Z_{0},\;t\in \left(T_{k-1},T_{k}\right],\;k=1,2,\ldots \;. \end{array}$$

Then, for $t\in (T_{k-1},T_{k}]$, we get the following estimation

(17) $$\begin{array}{c} ∥Z\left(t;t_{0},Z_{0},\left\{T_{k}\right\}\right)∥\leq ∥Z_{0}∥\prod_{i=1}^{k-1}\left(∥D\left(t_{k-i}\right)∥∥e^{C\left(t_{k-i}\right)t_{k-i}}∥\right)∥e^{C\left(t_{k}\right)\left(t-T_{k-1}\right)}∥\\ \leq ∥Z_{0}∥\prod_{i=1}^{k-1}\left(\alpha e^{||C\left(t_{k-i}\right){\left|\right|}_{2}t_{k-i}}\right)e^{||C\left(t_{k}\right)\left|\right|\left(t-T_{k-1}\right)}\\ \leq ∥Z_{0}∥\prod_{i=1}^{k-1}\left(\alpha e^{2Nt_{k-i}}\right)e^{2N(t-T_{k-1})}\\ \leq ∥Z_{0}∥\alpha^{k}e^{2N\left(\sum_{i=1}^{k-1}t_{k-i}+\left(t-T_{k-1}\right)\right)}=∥Y_{0}∥\alpha^{k}e^{2N(t-t_{0})}. \end{array}$$

The solutions $Z(t;t_{0},Z_{0},\left\{T_{k}\right\})$ generate continuous stochastic process $Z(t;t_{0},Z_{0},\left\{\tau_{k}\right\})$ that is defined by (16). It is a solution to initial value problem of impulsive differential equation with random moments of impulses (9). According to Proposition 2, Proposition 3, and inequality (17), we get

$$\begin{array}{cc} \; & E\left(∥Z(t;t_{0},Z_{0},\left\{\tau_{k}\right\})∥|S_{k}\left(t\right)\right)\leq ∥Z_{0}∥\alpha^{k}e^{2N(t-t_{0})}. \end{array}$$

Therefore, applying Corollary 1, we obtain

$$\begin{array}{cc} \; & E∥Z\left(t;t_{0},Z_{0},\left\{\tau_{k}\right\}\right)∥=\sum_{k=0}^{\infty }E\left(∥Z\left(t;t_{0},Z_{0},\left\{\tau_{k}\right\}\right)∥|S_{k}\left(t\right)\right)P\left(S_{k}\left(t\right)\right)\\ \; & \leq \sum_{k=0}^{\infty }∥Z_{0}∥\alpha^{k}e^{2N(t-t_{0})}e^{-\lambda (t-t_{0})}\frac{\lambda^{k}{\left(t-t_{0}\right)}^{k}}{k!}\\ \; & \leq ∥Z_{0}∥e^{\left(2N-\lambda \right)\left(t-t_{0}\right)}\sum_{k=0}^{\infty }\frac{{\left(\alpha \lambda \left(t-t_{0}\right)\right)}^{k}}{k!}=∥Z_{0}∥e^{\left(2N+\alpha \lambda -\lambda \right)\left(t-t_{0}\right)}. \end{array}$$

 □

**Remark** **3.** The inequalities (14) and (15) are satisfied only for $\omega_{i}\left(t\right),\;i=1,2,\ldots ,N$, such that $\omega_{i}\left(t\right)\neq 0$ for all i=1,2.…,N and $t\geq t_{0}$. Indeed, assume that there exist i=1,2…,N and $t^{*}\geq t_{0}$, such that $\omega_{i}\left(t^{*}\right)=0$. Then inequality (14) reduces to $|1-\sum_{j=1}^{N}a_{ij}\left(t^{*}\right)|<\frac{\alpha }{2N}$. If $1<\sum_{j=1}^{N}a_{ij}\left(t^{*}\right)$, then from (14) it follows that $1\leq \frac{\alpha (N-1)}{2N}$, i.e, $2\frac{N}{N-1}<\alpha$, which is not possible since α∈(0,1). Therefore, assume that $1\geq \sum_{j=1}^{N}a_{ij}\left(t^{*}\right)$ and $1-\sum_{j=1}^{N}a_{ij}\left(t^{*}\right)<\frac{\alpha }{2N}$. Hence $1<\frac{\alpha }{2N}+\sum_{j=1}^{N}a_{ij}\left(t^{*}\right)<\frac{\alpha }{2N}+\frac{\alpha (N-1)}{2N}=\frac{\alpha }{2}$ which is again a contradiction with assumption that α∈(0,1).

**Theorem** **2.** *If the assumptions of Theorem 1 are satisfied, then the leader-following consensus for multi-agent system (2) is reached asymptotically.*

**Proof.**  The claim follows from Theorem 1, Remark 1, the equality $∥Z_{0}∥=∥x^{0}∥$, and the inequalities

$$E∥x(t;t_{0},Z_{0},\left\{\tau_{k}\right\})∥\leq E∥Z(t;t_{0},Z_{0},\left\{\tau_{k}\right\})∥\leq ∥Z_{0}∥e^{\left(2N+\alpha \lambda -\lambda \right)\left(t-t_{0}\right)},$$

for i=1,2,…,N. □

According to Remark 3, condition (A1) is satisfied only in the case when a leader is available to each agent at any update random time. An interpretation of this situation can be the following. A leader can be viewed as the root node for the communication network; if there exists a directed path from the root to each agent (device), then all the agents can track the objective successfully. Since the leader can perceive more information in order to guide the whole group to complete the task (consensus), it seems to be reasonable to demand that he is available to each follower at any update random time.

## 5. Illustrative Examples

In this section, the numerical examples are given to verify the effectiveness of the proposed sufficient conditions for a multi-agent system to achieve asymptotically the leader-following consensus. In all examples, we set $t_{0}=0$ and consider a sequence of independent exponentially distributed random variables ${\left\{\tau_{k}\right\}}_{k=1}^{\infty }$ with parameter λ>0 (it will be defined later in each example) and the sequence of random variables ${\left\{\xi_{k}\right\}}_{k=0}^{\infty }$ defined by (1).

**Example** **1.** *Let us consider a system of three agents and the leader. In order to illustrate the meaningfulness of the studied model and the obtained results, we consider three cases.*

**Case 1.1.** *There is no information exchange between agents and the leader is not available.*

*The dynamics of agents are given by*

(18) $$\begin{array}{c} y_{r}^{\prime }\left(t\right)=0,\\ y_{1}^{\prime }\left(t\right)=-0.1\left(1+|\sin\left(t\right)|\right)y_{1}\left(t\right),\\ y_{2}^{\prime }\left(t\right)=-\left(\frac{0.9}{t+1}-0.8\cos^{2}\left(t\right)\right)y_{2}\left(t\right),\\ y_{3}^{\prime }\left(t\right)=-\left(0.4|\cos\left(t\right)|-0.1\right)y_{3}\left(t\right),\;\;t\geq 0. \end{array}$$

**Figure 1:**
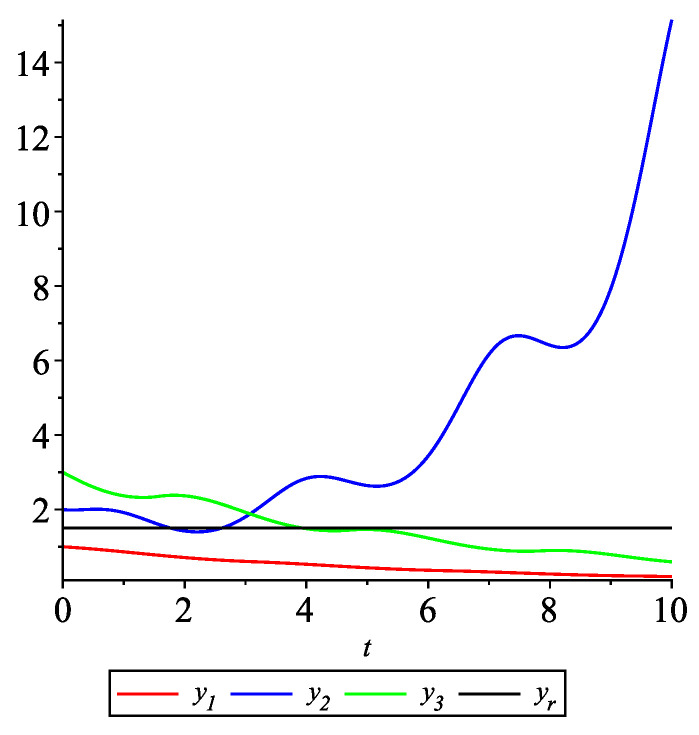
Example 1. Case 1.1. Graphs of the state trajectories $y_{i}\left(t\right),i=1,2,3$, of the agents and the leader $y_{r}$.

*Figure 1 shows the solution to system (18) with the initial values: $y_{1}^{0}=1$, $y_{2}^{0}=2$, $y_{3}^{0}=3$, $y_{r}^{0}=\frac{3}{2}$. From the graphs in Figure 1 it can be seen that the leader-following consensus is not reached.*

**Case 1.2.** *There is information exchange between agents (including the leader) occurring at random update times.*

*The dynamics between two update times of each agent and of the leader are given by (compare with (18)):*

(19) $$\begin{array}{c} y_{r}^{\prime }\left(t\right)=0,\\ y_{1}^{\prime }\left(t\right)=-0.1(1+|\sin\left(\tau_{k}\right)\left|\right)y_{1}\left(t\right),\\ y_{2}^{\prime }\left(t\right)=-\left(\frac{0.9}{\tau_{k}+1}-0.8\cos^{2}\left(\tau_{k}\right)\right)y_{2}\left(t\right),\\ y_{3}^{\prime }\left(t\right)=-(0.4|\cos\left(\tau_{k}\right)\left|-0.1\right)y_{3}\left(t\right)\;\;t\in \left(\xi_{k},\xi_{k+1}\right],\;\;\;k=0,1,2,\ldots . \end{array}$$

*The consensus control law at any update time $\xi_{k},\;k=1,2,\ldots ,$ is given by*

(20) $$\begin{array}{c} u_{1}\left(\xi_{k}\right)=-\frac{0.1\tau_{k}}{\tau_{k}+1}\left(y_{1}\left(\xi_{k}\right)-y_{2}\left(\xi_{k}\right)\right)-\left(1-0.05\cos^{2}\tau_{k}\right)\left(y_{1}\left(\xi_{k}\right)-y_{r}\left(\xi_{k}\right)\right),\\ u_{2}\left(\xi_{k}\right)=-\left(1-0.09\cos^{2}\tau_{k}\right)\left(y_{2}\left(\xi_{k}\right)-y_{r}\left(\xi_{k}\right)\right),\\ u_{3}\left(\xi_{k}\right)=-0.1\sin^{2}\left(\tau_{k}\right)\left(y_{3}\left(\xi_{k}\right)-y_{2}\left(\xi_{k}\right)\right)-\left(1-0.01\cos\left(\tau_{k}\right)\right)\left(y_{3}\left(\xi_{k}\right)-y_{r}\left(\xi_{k}\right)\right), \end{array}$$

*Hence,*

$$C\left(t\right)=\left[\begin{array}{cccccc} -0.1(1+|\sin(t)|) & 0.1(1+|\sin(t)|) & 0 & 0 & 0 & 0\\ 0 & 0 & 0 & 0 & 0 & 0\\ 0 & 0 & -\frac{0.9}{t+1} & \frac{0.9}{t+1} & 0 & 0\\ 0 & 0 & 0.8\cos^{2}\left(t\right) & -0.8\cos^{2}\left(t\right) & 0 & 0\\ 0 & 0 & 0 & 0 & -0.4(1+|\cos(t)|) & 0.4(1+|\cos(t)|)\\ 0 & 0 & 0 & 0 & 0.5 & -0.5 \end{array}\right]$$

*and*

$$D\left(t\right)=\left[\begin{array}{cccccc} 0 & 0 & 0 & 0 & 0 & 0\\ 0 & -\frac{0.1t}{t+1}+0.05\cos^{2}t & \frac{0.1t}{t+1} & 0 & 0 & 0\\ 0 & 0 & 0 & 0 & 0 & 0\\ 0 & 0 & 0 & 0.09\cos^{2}t & 0 & 0\\ 0 & 0 & 0 & 0 & 0 & 0\\ 0 & 0 & 0.1\sin^{2}\left(t\right) & 0 & 0 & -0.1\sin^{2}\left(t\right)+0.01\cos t \end{array}\right].$$

*Observe that, for α∈(0.6,1), Assumption* **(A1)** *of Theorem 1 is fulfilled. Let λ=45. Then, for α=0.7, Assumption* **(A2)** *of Theorem 1 holds. Therefore, by Theorem 2, the leader-following consensus for multi-agent system (19) with the consensus control law (20) at any update time is reached asymptotically.*

*To illustrate the behavior of the solutions of the model with impulses occurring at random times, we consider several sample path solutions. For $t_{0}=0$ we fix the initial values as follows: $y_{1}^{0}=1$, $y_{2}^{0}=2$, $y_{3}^{0}=3$, $y_{r}^{0}=\frac{3}{2}$, and choose different values of each random variable $\tau_{k},k=1,2,\ldots ,12$, in the following way:*

*(i)*  *$t_{1}=10,t_{2}=2,t_{3}=8,t_{4}=10,t_{5}=15,t_{6}=2,t_{7}=8,t_{8}=7,t_{9}=6,t_{10}=12,t_{11}=2,t_{12}=8$;*
*(ii)*  *$t_{1}=2,t_{2}=12,t_{3}=10,t_{4}=6,t_{5}=5,t_{6}=2,t_{7}=7,t_{8}=6,t_{9}=5,t_{10}=10,t_{11}=7,t_{12}=18$;*
*(iii)*  *$t_{1}=3,t_{2}=9,t_{3}=11,t_{4}=7,t_{5}=5,t_{6}=9,t_{7}=6,t_{8}=7,t_{9}=2,t_{10}=6,t_{11}=15,t_{12}=10$;*
*(iv)*  *$t_{1}=7,t_{2}=5,t_{3}=8,t_{4}=10,t_{5}=5,t_{6}=7,t_{7}=4,t_{8}=11,t_{9}=8,t_{10}=6,t_{11}=9,t_{12}=10$.*

**Figure 2:**
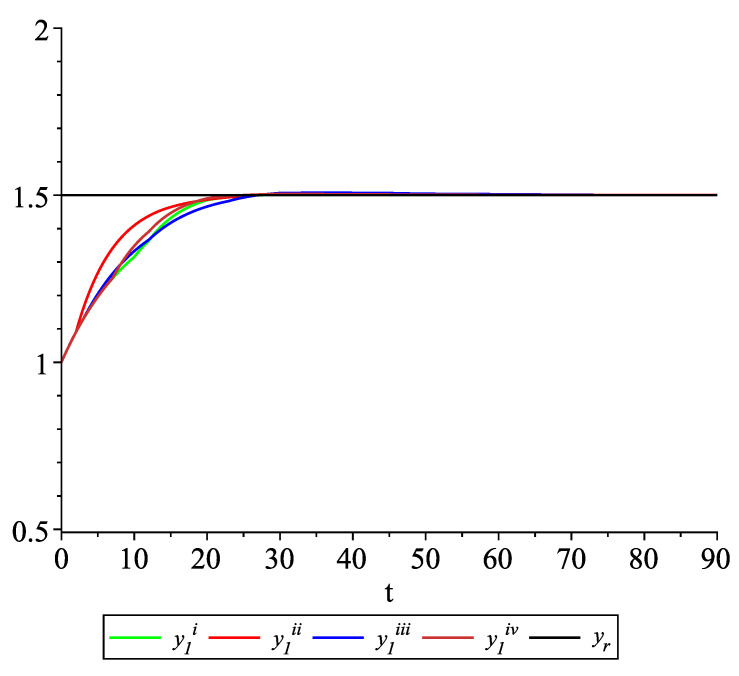
Example 1. Case 1.2. Graphs of the state trajectory $y_{1}\left(t\right)$ of the first agent for various values of random variables $\tau_{k}$.

**Figure 3:**
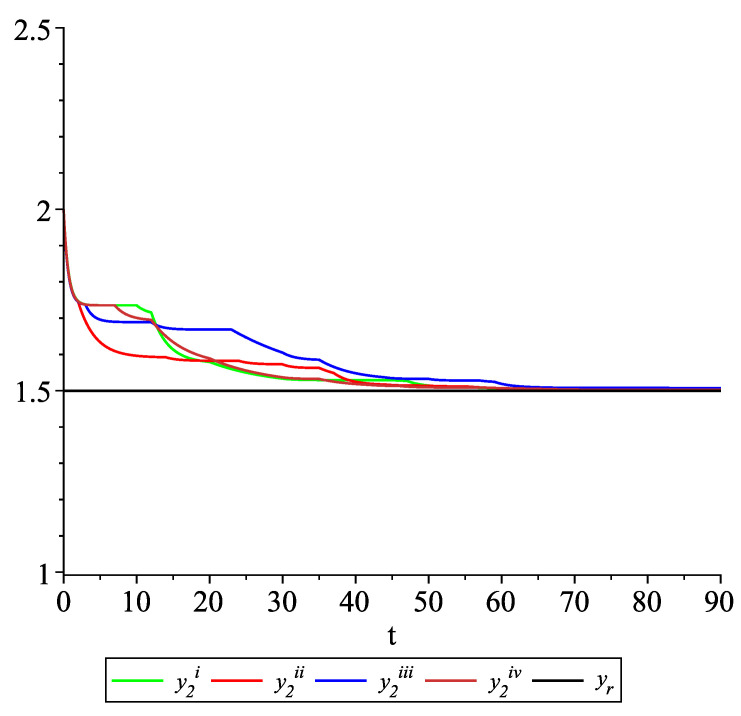
Example 1. Case 1.2. Graphs of the state trajectory $y_{2}\left(t\right)$ of the second agent for various values of random variables $\tau_{k}$.

**Figure 4:**
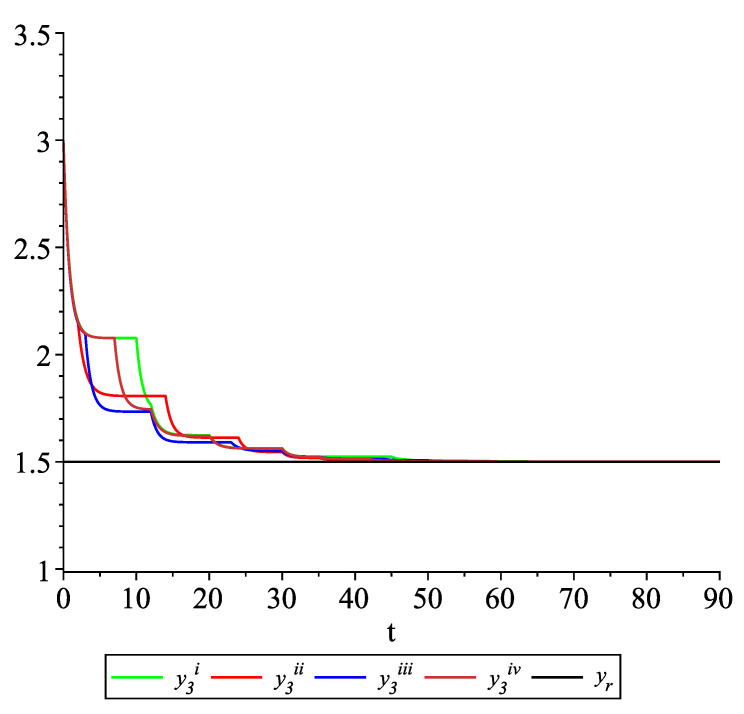
Example 1. Case 1.2. Graphs of the state trajectory $y_{3}\left(t\right)$ of the third agent for various values of random variables $\tau_{k}$.

*Clearly, the leader state is $y_{r}\left(t\right)\equiv 1.5$. For each value of the random variables (i)–(iv) we get the system of impulsive differential equations with fixed points of impulses of type (11) with N=3 and matrices C(t), D(t) given above. Figure 2, Figure 3 and Figure 4 present the state trajectories of the leader $y_{r}\left(t\right)$ and agent $y_{1}\left(t\right),y_{2}\left(t\right)$ and $y_{3}\left(t\right)$, respectively. Apparently, it is visible that the leader-following consensus is reached for all considered sample path solutions.*

**Case 1.3.** *At any update random time, only the leader is available to each agent and there is no information exchange between agents.*

*The dynamics between two update times of each agent are given by (19), and at update time $\xi_{k},\;k=1,2,\ldots ,$ the following control law is applied:*

(21) $$\begin{array}{c} u_{1}\left(\xi_{k}\right)=-\left(1-0.09\cos^{2}\left(\tau_{k}\right)\right)\left(y_{1}\left(\xi_{k}\right)-y_{r}\left(\xi_{k}\right)\right),\\ u_{2}\left(\xi_{k}\right)=-\left(1+0.09\sin\left(\tau_{k}\right)\right)\left(y_{2}\left(\xi_{k}\right)-y_{r}\left(\xi_{k}\right)\right),\\ u_{3}\left(\xi_{k}\right)=-\left(1-\frac{0.1\tau_{k}}{\tau_{k}+1}\right)\left(y_{3}\left(\xi_{k}\right)-y_{r}\left(\xi_{k}\right)\right). \end{array}$$

*Therefore,*

$$D\left(t\right)=\left[\begin{array}{cccccc} 0 & 0 & 0 & 0 & 0 & 0\\ 0 & 0.09\cos^{2}t & 0 & 0 & 0 & 0\\ 0 & 0 & 0 & 0 & 0 & 0\\ 0 & 0 & 0 & -0.09\sin t & 0 & 0\\ 0 & 0 & 0 & 0 & 0 & 0\\ 0 & 0 & 0 & 0 & 0 & \frac{0.1t}{t+1} \end{array}\right]$$

*and C(t) is the same as in Case 1.2. It is easy to check that, for α=0.7 and λ=45, assumptions* **(A1)** *and* **(A2)** *are fulfilled. According to Theorem 2, the leader-following consensus for multi-agent system (19) with the consensus control law (21) at any update time is reached asymptotically.*

**Figure 5:**
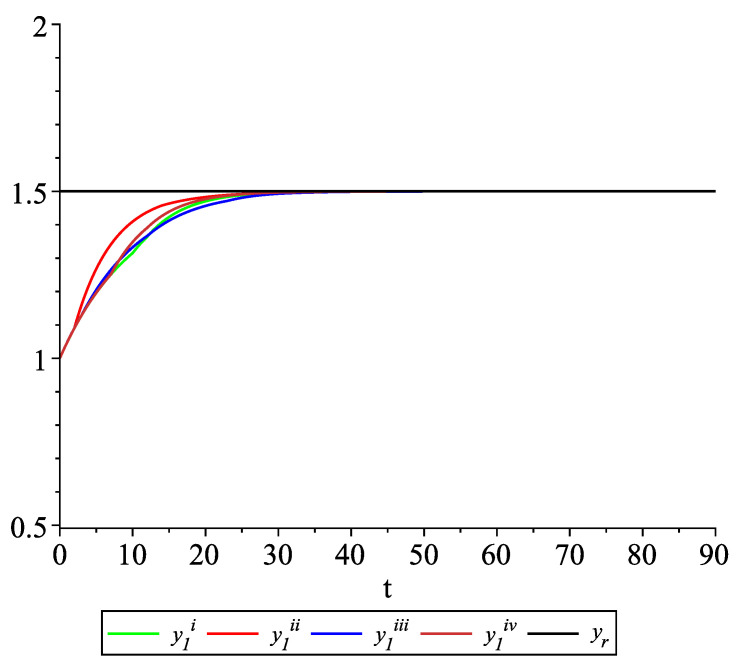
Example 1. Case 1.3. Graphs of the state trajectory $y_{1}\left(t\right)$ of the first agent for various values of random variables $\tau_{k}$.

**Figure 6:**
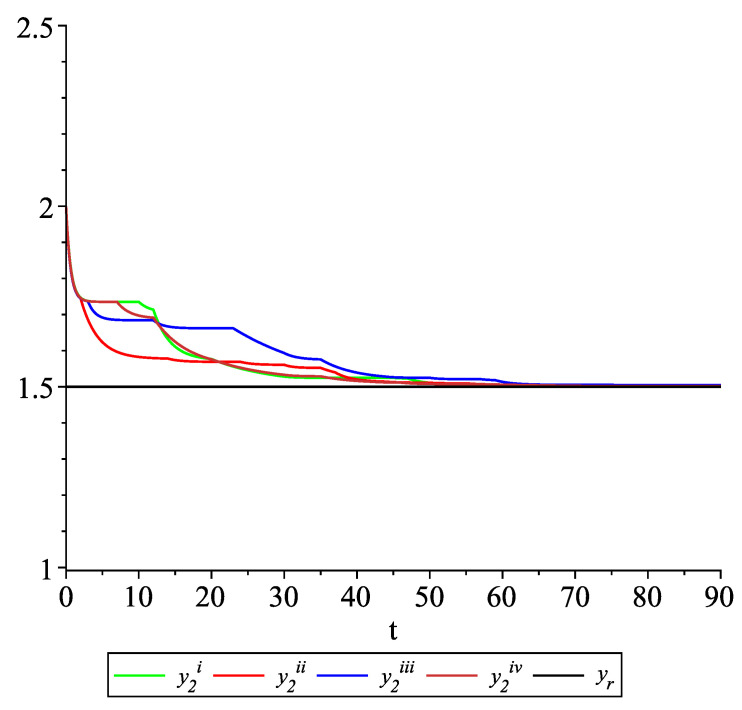
Example 1. Case 1.3. Graphs of the state trajectory $y_{2}\left(t\right)$ of the second agent for various values of random variables $\tau_{k}$.

**Figure 7:**
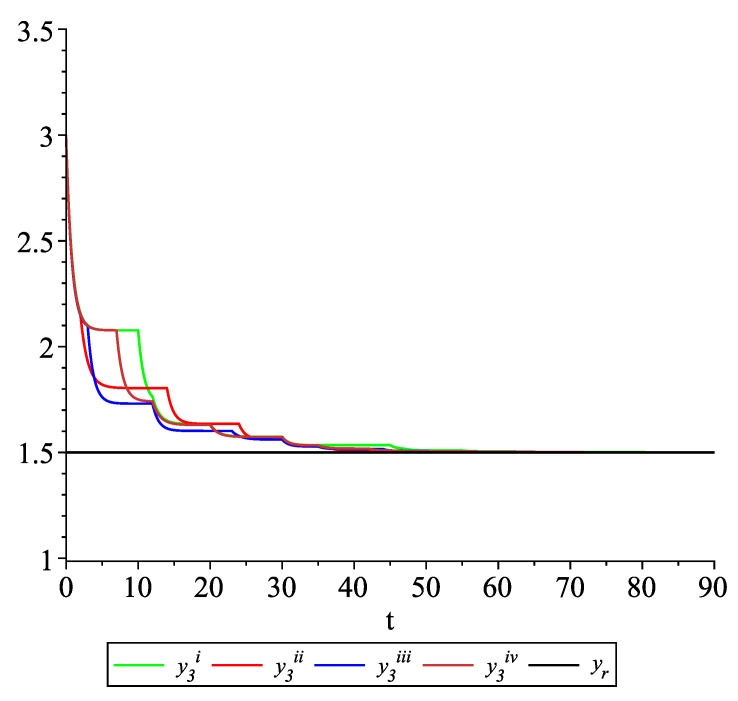
Example 1. Case 1.3. Graphs of the state trajectory $y_{3}\left(t\right)$ of the third agent for various values of random variables $\tau_{k}$.

*To illustrate the behavior of the solutions of the model with impulses occurring at random times, we consider sample path solutions with the same data as in Case 1.2. Figure 5, Figure 6 and Figure 7 present the state trajectories of the leader $y_{r}\left(t\right)$ and agent $y_{1}\left(t\right),y_{2}\left(t\right)$ and $y_{3}\left(t\right)$, respectively. Apparently, it is visible that the leader-following consensus is reached in all considered sample path solutions.*

**Example** **2.** *Let the system consist of four agents and the leader. In order to illustrate the meaningfulness of the studied model and the obtained results, we consider four cases.*

**Case 2.1.** *There is no information exchange between agents and the leader is not available.*

*The dynamics of agents are given by*

(22) $$\begin{array}{c} y_{r}^{\prime }\left(t\right)=0,\\ y_{1}^{\prime }\left(t\right)=-\left(0.4-0.1\sin^{2}\left(t\right)\right)y_{1}\left(t\right),\\ y_{2}^{\prime }\left(t\right)=-\left(0.3{\left(1.01\right)}^{-t}-0.1\cos^{2}\left(t\right)\right)y_{2}\left(t\right),\\ y_{3}^{\prime }\left(t\right)=\frac{-0.5}{t+1}y_{3}\left(t\right),\\ y_{4}^{\prime }\left(t\right)=-\left(\frac{0.3}{t+1}-0.1\cos^{2}\left(t\right)\right)y_{4}\left(t\right), \end{array}$$

**Figure 8:**
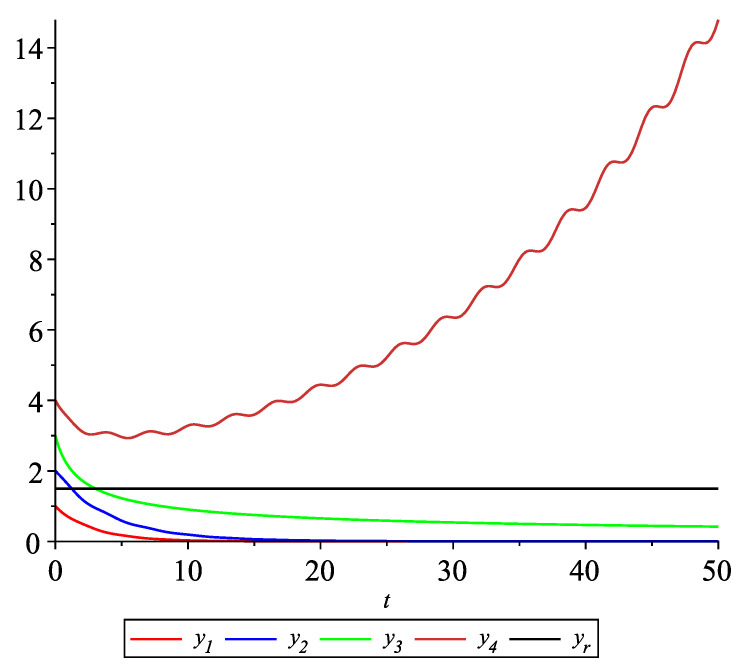
Example 2. Case 2.1. Graphs of the state trajectories $y_{i}\left(t\right),i=1,2,3$ of the agents and the leader $y_{r}$.

*Figure 8 shows the solution to system (22) with the initial values: $y_{1}^{0}=1$, $y_{2}^{0}=2$, $y_{3}^{0}=3$, $y_{4}^{0}=4$, $y_{r}^{0}=\frac{3}{2}$. It is visible that the leader-following consensus is not reached.*

**Case 2.2.** *There is information exchange between agents occurring at random update times and the leader is available for agents.*

*The dynamics between two update times of each agent and of the leader are given by*

(23) $$\begin{array}{c} y_{r}^{\prime }\left(t\right)=0,\\ y_{1}^{\prime }\left(t\right)=-\left(0.4-0.1\sin^{2}\left(\tau_{k}\right)\right)y_{1}\left(t\right),\\ y_{2}^{\prime }\left(t\right)=-\left(0.3{\left(1.01\right)}^{-\tau_{k}}-0.9\cos^{2}\left(\tau_{k}\right)\right)y_{2}\left(t\right),\\ y_{3}^{\prime }\left(t\right)=\frac{-0.5}{\tau_{k}+1}y_{3}\left(t\right),\\ y_{4}^{\prime }\left(t\right)=-\left(\frac{0.3}{\tau_{k}+1}-0.1\cos^{2}\left(\tau_{k}\right)\right)y_{4}\left(t\right),\;\;t\in \left(\xi_{k},\xi_{k+1}\right],\;\;\;k=0,1,2,\ldots . \end{array}$$

*At update time $\xi_{k},\;k=1,2,\ldots ,$ the following control law is applied:*

(24) $$\begin{array}{c} u_{1}\left(\xi_{k}\right)=-0.1\sin^{2}\left(\tau_{k}\right)\left(y_{1}\left(\xi_{k}\right)-y_{2}\left(\xi_{k}\right)\right)-0.9\left(y_{1}\left(\xi_{k}\right)-y_{r}\left(\xi_{k}\right)\right),\\ u_{2}\left(\xi_{k}\right)=-0.1e^{-\tau_{k}^{2}}\left(y_{2}\left(\xi_{k}\right)-y_{1}\left(\xi_{k}\right)\right)-\left(y_{2}\left(\xi_{k}\right)-y_{r}\left(\xi_{k}\right)\right),\\ u_{3}\left(\xi_{k}\right)=-\left(y_{3}\left(\xi_{k}\right)-y_{r}\left(\xi_{k}\right)\right),\\ u_{4}\left(\xi_{k}\right)=-0.06|\sin\left(\tau_{k}\right)|\left(y_{4}\left(\xi_{k}\right)-y_{1}\left(\xi_{k}\right)\right)-0.06\left|\cos\left(\tau_{k}\right)\right|\left(y_{4}\left(\xi_{k}\right)-y_{2}\left(\xi_{k}\right)\right)\\ \;\;\;\;\;\;\;\;\;\;\;\;\;\;\;\;\;\;\;\;\;\;-\left(y_{4}\left(\xi_{k}\right)-y_{r}\left(\xi_{k}\right)\right),\;\;\;k=1,2,\ldots . \end{array}$$

*In this case, we have*

$$D\left(t\right)=\left[\begin{array}{cccccccc} 0 & 0 & 0 & 0 & 0 & 0 & 0 & 0\\ 0 & -0.1\sin^{2}\left(t\right)+0.1 & 0.1\sin^{2}\left(t\right) & 0 & 0 & 0 & 0 & 0\\ 0 & 0 & 0 & 0 & 0 & 0 & 0 & 0\\ 0.1e^{-t^{2}} & 0 & 0 & -0.1e^{-t^{2}} & 0 & 0 & 0 & 0\\ 0 & 0 & 0 & 0 & 0 & 0 & 0 & 0\\ 0 & 0 & 0 & 0 & 0 & 0 & 0 & 0\\ 0 & 0 & 0 & 0 & 0 & 0 & 0 & 0\\ 0.06|\sin(t)| & 0 & 0.06|\cos(t)| & 0 & 0 & 0 & 0 & -0.06(|\sin(t)|+|\cos(t)|) \end{array}\right]$$

*and*

$$C\left(t\right)=\left[\begin{array}{cccccccc} -0.4 & 0.4 & 0 & 0 & 0 & 0 & 0 & 0\\ 0.1\sin^{2}\left(t\right) & -0.1\sin^{2}\left(t\right) & 0 & 0 & 0 & 0 & 0 & 0\\ 0 & 0 & -0.3{\left(1.01\right)}^{-t} & 0.3{\left(1.01\right)}^{-t} & 0 & 0 & 0 & 0\\ 0 & 0 & 0.1\cos^{2}\left(t\right) & -0.1\cos^{2}\left(t\right) & 0 & 0 & 0 & 0\\ 0 & 0 & 0 & 0 & -\frac{0.5}{t+1} & \frac{0.5}{t+1} & 0 & 0\\ 0 & 0 & 0 & 0 & 0 & 0 & 0 & 0\\ 0 & 0 & 0 & 0 & 0 & 0 & -\frac{0.3}{t+1} & \frac{0.3}{t+1}\\ 0 & 0 & 0 & 0 & 0 & 0 & 0.1\cos^{2}\left(t\right) & -0.1\cos^{2}\left(t\right) \end{array}\right].$$

*Hence, for α∈(0.8,1), Assumption* **(A1)** *of Theorem 1 is fulfilled. Let λ=55. Then, for α=0.85, Assumption* **(A2)** *of Theorem 1 holds. Therefore, by Theorem 2, the leader-following consensus for multi-agent system (23) with the consensus control law (24) at any update time is reached asymptotically.*

*To illustrate the behavior of the solutions of the model with impulses occurring at random times, we consider several sample path solutions. For $t_{0}=0$ we fix the initial values as follows: $y_{1}^{0}=1$, $y_{2}^{0}=2$, $y_{3}^{0}=3$, $y_{4}^{0}=4$$y_{r}^{0}=\frac{3}{2}$; and choose different values of each random variable $\tau_{k},k=1,2,\ldots ,12$, in the following way:*

*(i)*  *$t_{1}=10,t_{2}=2,t_{3}=8,t_{4}=10,t_{5}=15,t_{6}=2,t_{7}=8,t_{8}=7,t_{9}=6,t_{10}=12,t_{11}=2,t_{12}=8$;*
*(ii)*  *$t_{1}=2,t_{2}=12,t_{3}=10,t_{4}=6,t_{5}=5,t_{6}=2,t_{7}=7,t_{8}=6,t_{9}=5,t_{10}=10,t_{11}=7,t_{12}=18$;*
*(iii)*  *$t_{1}=3,t_{2}=9,t_{3}=11,t_{4}=7,t_{5}=5,t_{6}=9,t_{7}=6,t_{8}=7,t_{9}=2,t_{10}=6,t_{11}=15,t_{12}=10$;*
*(iv)*  *$t_{1}=7,t_{2}=\pi ,t_{3}=8,t_{4}=\pi /2,t_{5}=5,t_{6}=2\pi ,t_{7}=4,t_{8}=11,t_{9}=3\pi /2,t_{10}=4\pi ,t_{11}=17,t_{12}=10$.*

*Clearly, the leader state is $y_{r}\left(t\right)\equiv 1.5$.*

**Figure 9:**
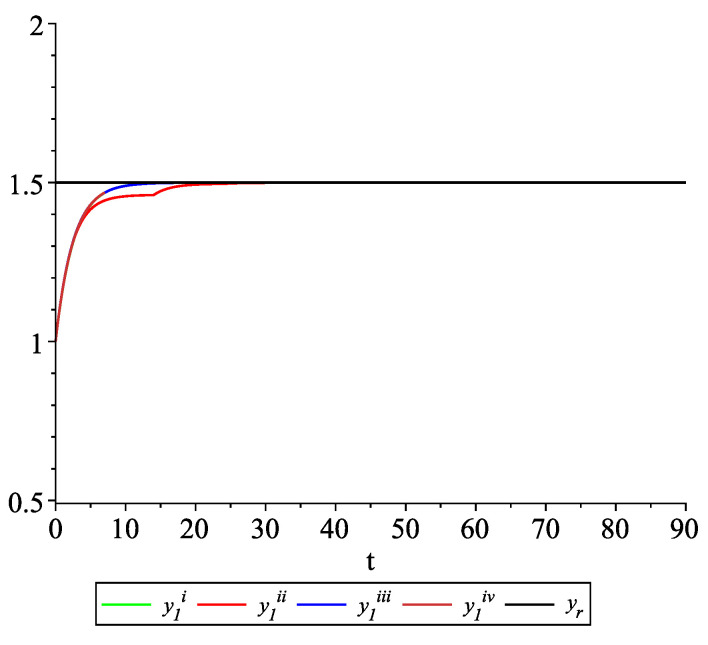
Example 2. Case 2.2. Graphs of the state trajectory $y_{1}\left(t\right)$ of the first agent for various values of random variables $\tau_{k}$.

**Figure 10:**
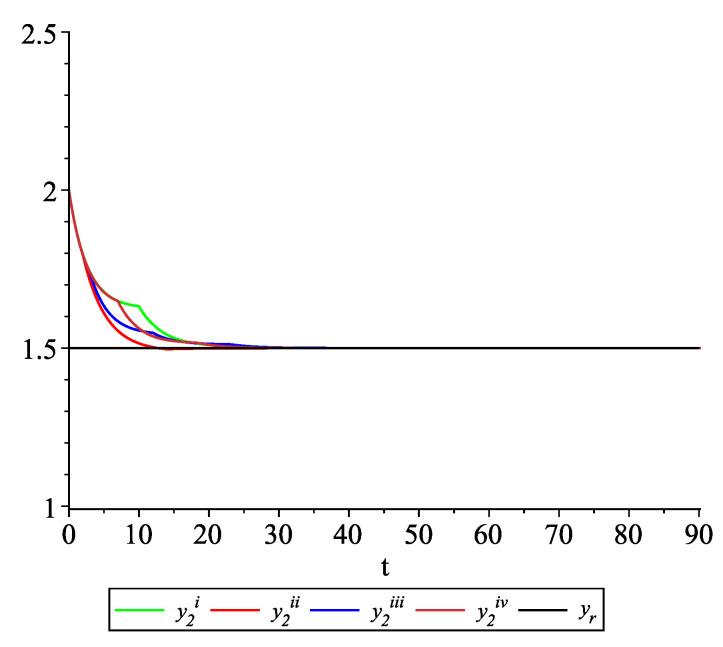
Example 2. Case 2.2. Graphs of the state trajectory $y_{2}\left(t\right)$ of the second agent for various values of random variables $\tau_{k}$.

**Figure 11:**
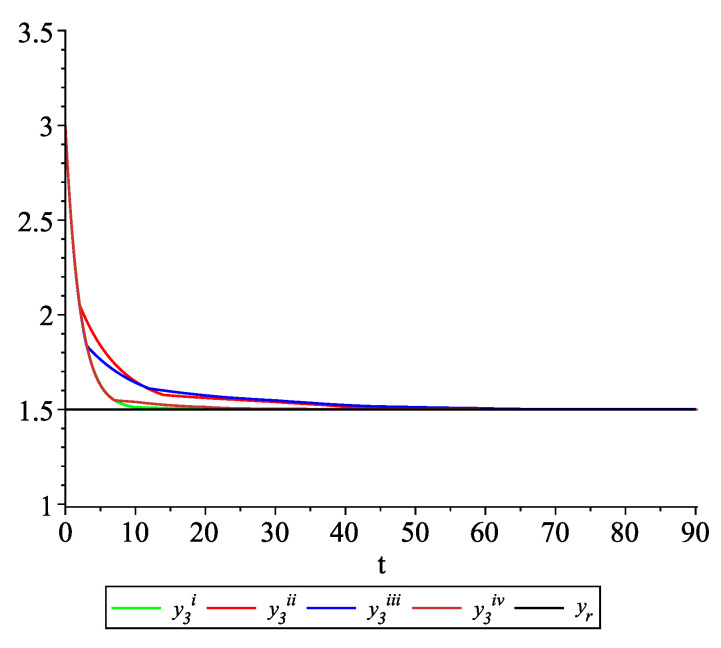
Example 2. Case 2.2. Graphs of the state trajectory $y_{3}\left(t\right)$ of the third agent for various values of random variables $\tau_{k}$.

**Figure 12:**
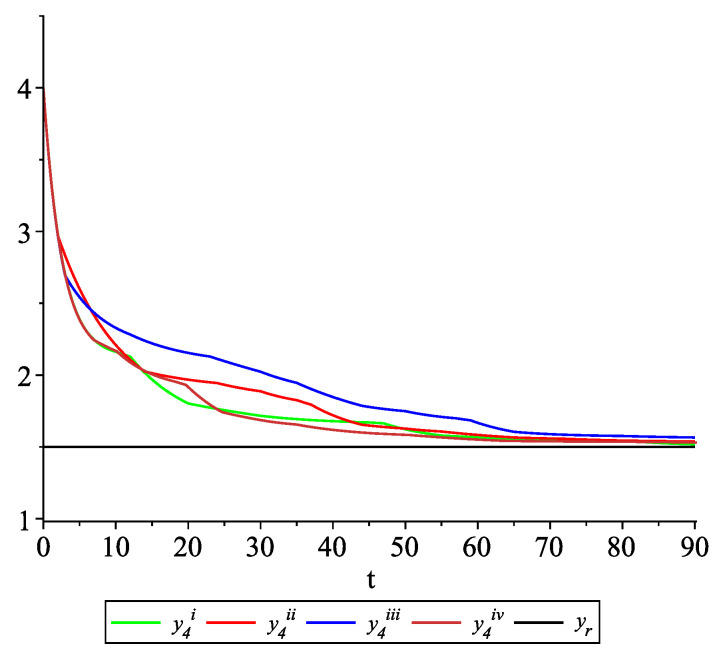
Example 2. Case 2.2. Graphs of the state trajectory $y_{4}\left(t\right)$ of the fourth agent for various values of random variables $\tau_{k}$.

*Figure 9, Figure 10, Figure 11 and Figure 12 present the state trajectories of the leader $y_{r}\left(t\right)$ and agent $y_{1}\left(t\right),y_{2}\left(t\right),y_{3}\left(t\right)$, and $y_{4}\left(t\right)$, respectively. Apparently, it is visible that the leader-following consensus is reached for all considered sample path solutions.*

**Case 2.3.** *There is information exchange between agents occurring at random update times but the leader is not available for agents.*

*The dynamics between two update times of each agent are given by (23), and at update time $\xi_{k},\;k=1,2,\ldots ,$ the following control law is applied:*

$$\begin{array}{cc} \; & u_{1}\left(\xi_{k}\right)=-0.1\sin^{2}\left(\tau_{k}\right)\left(y_{1}\left(\xi_{k}\right)-y_{2}\left(\xi_{k}\right)\right),\\ \; & u_{2}\left(\xi_{k}\right)=-0.1e^{-\tau_{k}^{2}}\left(y_{2}\left(\xi_{k}\right)-y_{1}\left(\xi_{k}\right)\right),\\ \; & u_{3}\left(\xi_{k}\right)=0,\\ \; & u_{3}\left(\xi_{k}\right)=-0.06|\sin\left(\tau_{k}\right)|\left(y_{4}\left(\xi_{k}\right)-y_{1}\left(\xi_{k}\right)\right)-0.06\left|\cos\left(\tau_{k}\right)\right|\left(y_{4}\left(\xi_{k}\right)-y_{2}\left(\xi_{k}\right)\right). \end{array}$$

*In this case, $\omega_{i}\left(t\right)\equiv 0,\;t\geq 0,\;i=1,2,3,4$, and inequalities (13) and (15) are satisfied. According to observation in Remark 3, Assumption (A1) is not fulfilled.*

**Figure 13:**
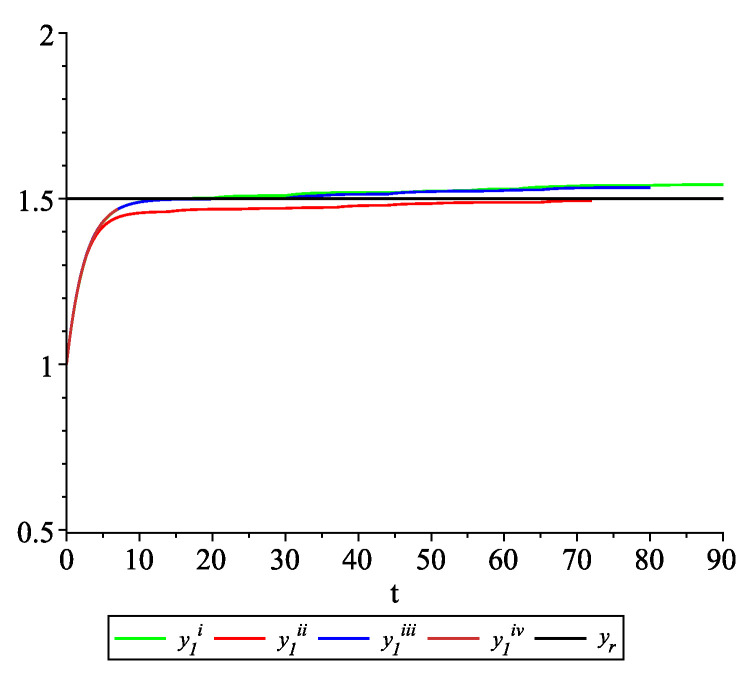
Example 2. Case 2.3. Graphs of the state trajectory $y_{1}\left(t\right)$ of the first agent for various values of random variables $\tau_{k}$.

**Figure 14:**
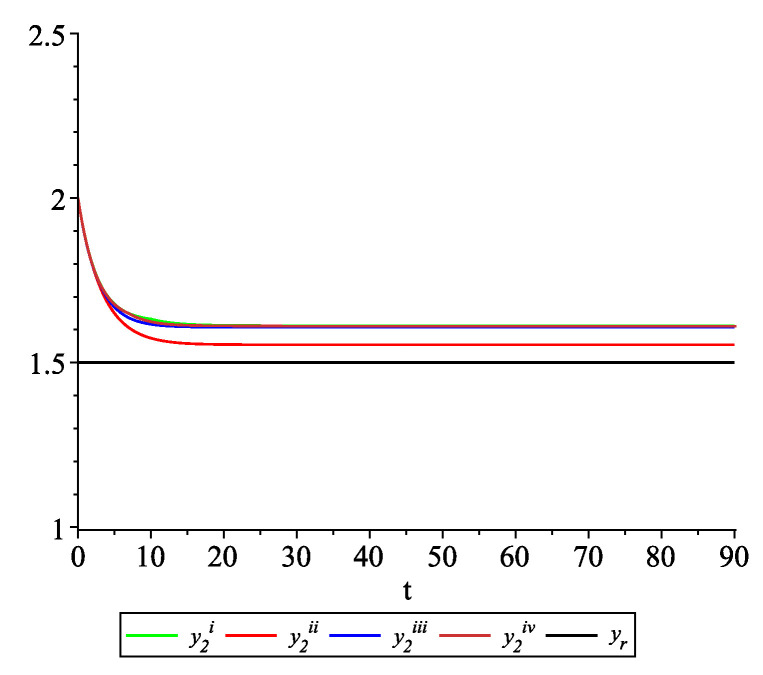
Example 2. Case 2.3. Graphs of the state trajectory $y_{2}\left(t\right)$ of the second agent for various values of random variables $\tau_{k}$.

**Figure 15:**
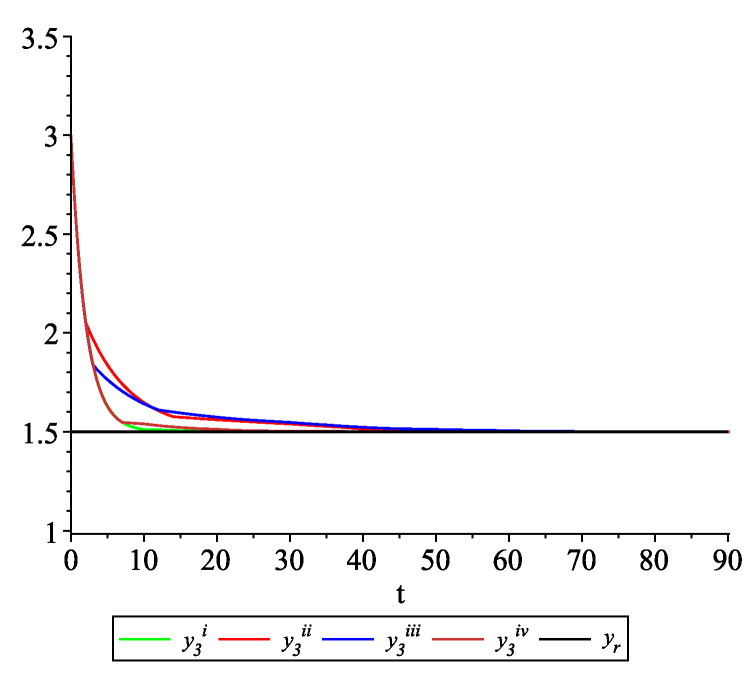
Example 2. Case 2.3. Graphs of the state trajectory $y_{3}\left(t\right)$ of the third agent for various values of random variables $\tau_{k}$.

**Figure 16:**
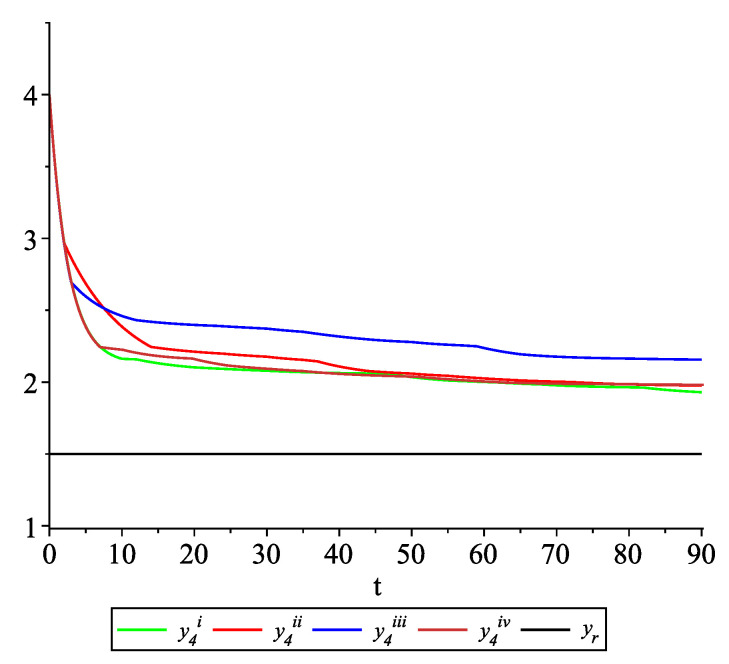
Example 2. Case 2.3. Graphs of the state trajectory $y_{4}\left(t\right)$ of the fourth agent for various values of random variables $\tau_{k}$.

*To illustrate the behavior of the solutions of the model with impulses occurring at random times, we fix λ=55 and consider sample path solutions with the same data as in Case 2.2. Figure 13, Figure 14, Figure 15 and Figure 16 present the state trajectories of the leader $y_{r}\left(t\right)$ and agent $y_{1}\left(t\right),y_{2}\left(t\right),y_{3}\left(t\right)$ and $y_{4}\left(t\right)$, respectively. Observe that the leader-following consensus is not reached in all considered sample path solutions.*

**Case 2.4.** *The leader is not available to one agent at all update times.*

*The dynamics between two update times of each agent are given by (23), and at update time $\xi_{k},\;k=1,2,\ldots ,$ the control law is applied:*

$$\begin{array}{cc} \; & u_{1}\left(\xi_{k}\right)=-0.1\sin^{2}\left(\tau_{k}\right)\left(y_{1}\left(\xi_{k}\right)-y_{2}\left(\xi_{k}\right)\right)-0.9\left(y_{1}\left(\xi_{k}\right)-y_{r}\left(\xi_{k}\right)\right),\\ \; & u_{2}\left(\xi_{k}\right)=-0.1e^{-\tau_{k}^{2}}\left(y_{2}\left(\xi_{k}\right)-y_{1}\left(\xi_{k}\right)\right),\\ \; & u_{3}\left(\xi_{k}\right)=-\left(y_{3}\left(\xi_{k}\right)-y_{r}\left(\xi_{k}\right)\right),\\ \; & u_{4}\left(\xi_{k}\right)=-0.06|\sin\left(\tau_{k}\right)|\left(y_{4}\left(\xi_{k}\right)-y_{1}\left(\xi_{k}\right)\right)-0.06\left|\cos\left(\tau_{k}\right)\right|\left(y_{4}\left(\xi_{k}\right)-y_{2}\left(\xi_{k}\right)\right)\\ \; & \;\;\;\;\;\;\;\;\;\;\;\;\;\;\;\;\;\;\;\;\;\;-\left(y_{4}\left(\xi_{k}\right)-y_{r}\left(\xi_{k}\right)\right),\;\;\;k=1,2,\ldots . \end{array}$$

*Since $\omega_{2}\left(t\right)\equiv 0$, by Remark 3, Assumption (* **A1** *) is not fulfilled.*

*To illustrate the behavior of the solutions of the model with impulses occurring at random times, we consider sample path solutions with the same data as in Case 2.2.*

**Figure 17:**
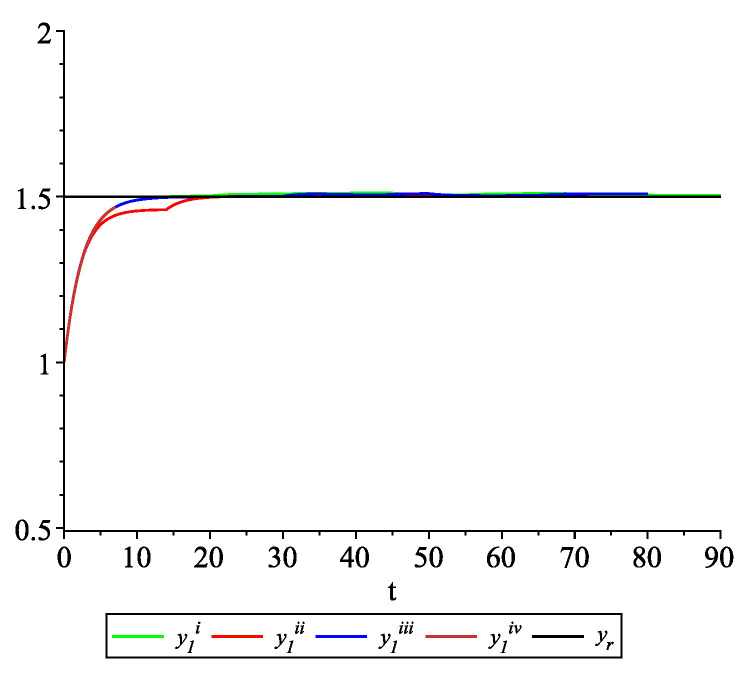
Example 2. Case 2.4. Graphs of the state trajectory $y_{1}\left(t\right)$ of the first agent for various values of random variables $\tau_{k}$.

**Figure 18:**
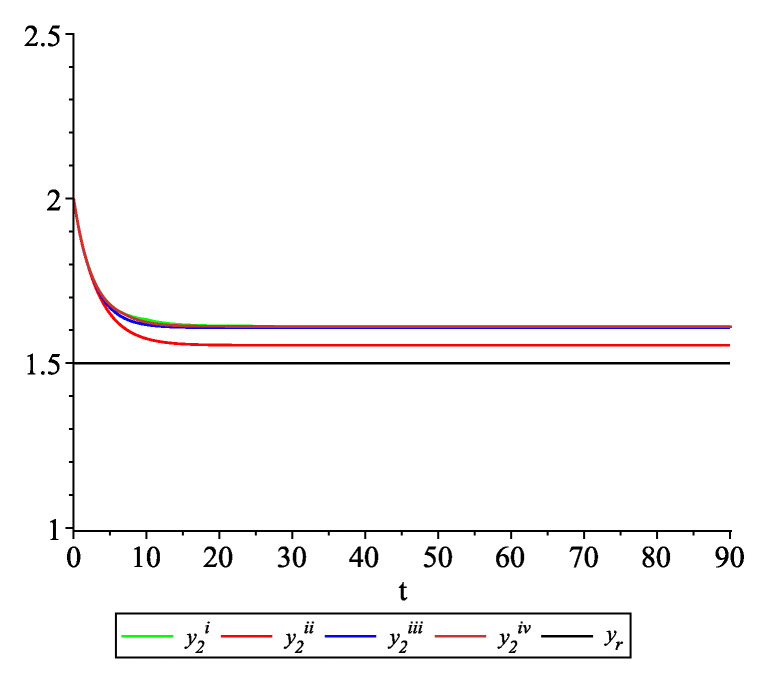
Example 2. Case 2.4. Graphs of the state trajectory $y_{2}\left(t\right)$ of the second agent for various values of random variables $\tau_{k}$.

**Figure 19:**
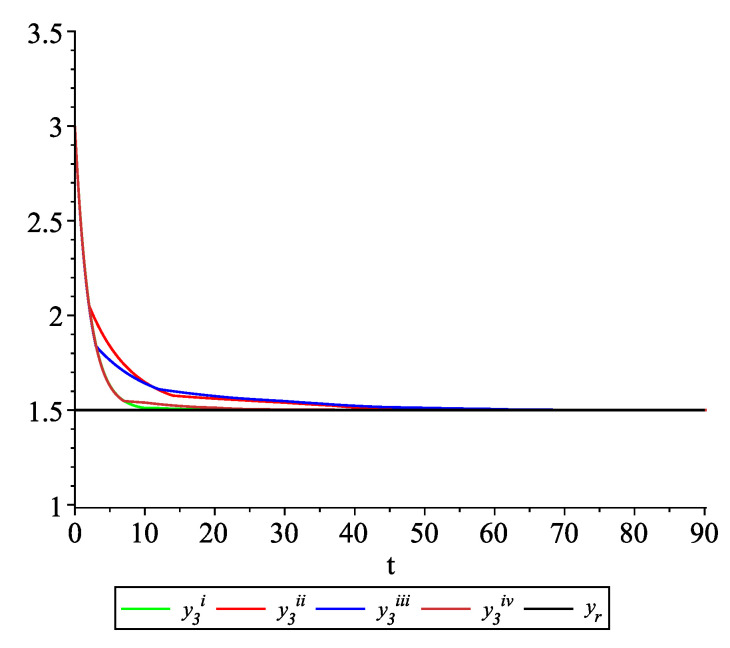
Example 2. Case 2.4. Graphs of the state trajectory $y_{3}\left(t\right)$ of the third agent for various values of random variables $\tau_{k}$.

**Figure 20:**
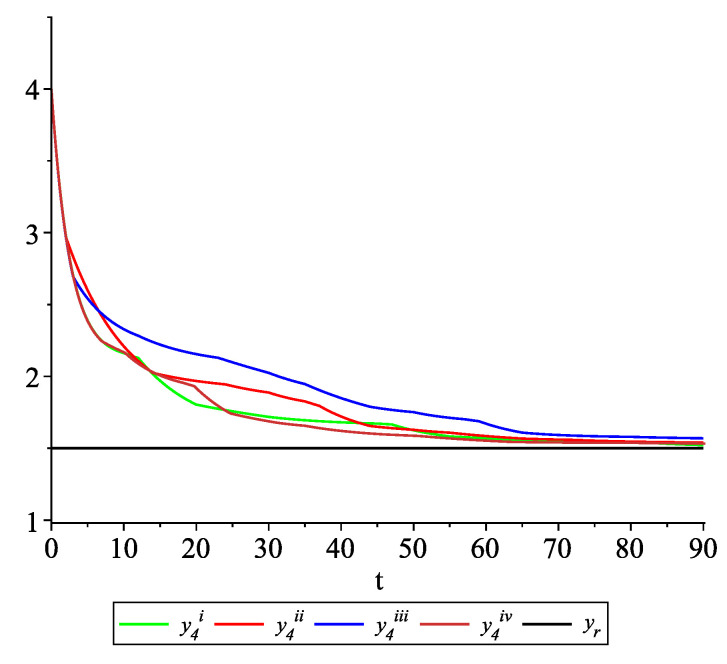
Example 2. Case 2.4. Graphs of the state trajectory $y_{4}\left(t\right)$ of the fourth agent for various values of random variables $\tau_{k}$.

*Figure 17, Figure 18, Figure 19 and Figure 20 present the state trajectories of 4 agents and the leader, respectively. Observe that the leader-following consensus is not reached in all considered sample path solutions. It is visible in Figure 18 where the graphs of the state trajectory of the second agent for various values of random variables are presented. This shows the importance of Assumption* (**A1**). *However, we emphasize that in the model considered in this paper the information exchange between agents is possible only at discrete random update times and the waiting time between two consecutive updates is exponentially distributed (similarly to queuing theory). Of course, in general, it is obvious that if the leader is continuously available for agents, then the leader-following consensus is reached. But in this paper, we consider the situation when the leader is available just from time to time at random times (so he is not available continuously). We deliver conditions under which, in spite of lack of this continuous information flow from the leader to agents, the leader-following consensus is still reached.*

Both examples illustrate that the interaction between the leader and the other agents only at random update times changes significantly the behavior of the agents. If conditions (**A1**) and (**A2**) are satisfied, then the leader-following consensus is reached in multi-system (2).

## 6. Conclusions

The leader-following consensus problem is a key point in the analysis of dynamic multi-agent networks. In this paper, we considered the situation when agents exchanged information only at discrete-time instants that occurred randomly. The proposed control law was distributed, in the sense that only information from neighboring agents was included, which implied that the control law was applied only at update times that occurred randomly. In the cases wherein the random update times were equal to the initially given times, our model was reduced to a continuous-time multi-agent system with discrete-time communications studied in [25]. The main difference between our model and the previous approaches was that we considered a sequence of update times as a sequence of random variables. Besides, unlike in other investigations, the waiting time between two consecutive updates was exponentially distributed. This was motivated by the most useful distribution in queuing theory. The presence of randomly occurring update times required using results from the probability theory and the theory of differential equations with impulses in order to describe our proposed solution to the considered multi-agent system. We provided conditions on the control law that ensured asymptotic leader-following consensus in the sense of the expected value of a stochastic process. This work may be treated as the first step towards the analysis of consensus problems of multi-agents with discrete updates at random times. For example, one of the possible problems to be investigated in the future is to deliver conditions under which the consensus is achieved in the multi-agent system with discrete updates at random times in spite of denial-of-service attacks or for systems with double-integrator dynamics. Another important and interesting issue is to work out a model of a real-world system of agents and to apply our theoretical results. For this purpose, we have to develop or adapt the existing numerical procedures for simulating the evolution of a system with a greater number of agents. This problem is currently under investigation.
